# Air-conditioning adoption and electricity demand highlight climate change mitigation–adaptation tradeoffs

**DOI:** 10.1038/s41598-023-31469-z

**Published:** 2023-03-17

**Authors:** Francesco Pietro Colelli, Ian Sue Wing, Enrica De Cian

**Affiliations:** 1 Dept. of Economics, Cannaregio, University of Venice Ca’ Foscari, Venice, 30120 Italy; 2 CMCC Foundation, Venice, 10587 Italy; 3 Department of Earth & Environment, 685 Commonwealth Ave., Boston, MA 02215 USA

**Keywords:** Climate-change adaptation, Climate-change impacts

## Abstract

We elucidate mid-century climate change impacts on electricity demand accounting for endogenous adoption of residential air-conditioning (AC) in affluent, cooler countries in Europe, and in poorer, hotter states in India. By 2050, in a high-warming scenario (SSP585) AC prevalence grows twofold in Europe and fourfold in India, reaching around 40% in both regions. We document a mitigation-adaptation tradeoff: AC expansion reduces daily heat exposures by 150 million and 3.8 billion person degree-days (PDDs), but increases annual electricity demand by 34 TWh and 168 TWh in Europe and India, respectively (corresponding to 2% and 15% of today’s consumption). The increase in adoption and use of AC would result in an additional 130 MMTCO2, of which 120 MMTCO2 in India alone, if the additional electricity generated were produced with today’s power mix. The tradeoff varies geographically and across income groups: a one PDD reduction in heat exposure in Europe versus India necessitates five times more electricity (0.53 kWh vs 0.1 kWh) and two times more emissions (0.16 kgCO$_2$ vs 0.09 kgCO$_2$), on average. The decomposition of demand drivers offers important insights on how such tradeoff can be moderated through policies promoting technology-based and behavioral-based adaptation strategies.

## Introduction

Air conditioning (AC) is a mature and widely available technology that uses electricity to maintain comfortable indoor environments during ambient high temperature periods. While institutional factors have led to historically high AC prevalence in the United States, adoption has increased in both Europe and the developing world^1–5^. Given projected future growth of cooling demand^6,7^ and AC ownership worldwide^4,8^, a major concern is that climate change-driven increases in the frequency and intensity of high temperature extremes^9^ will amplify electricity demand to levels that exceed power systems’ capacity, adversely affecting reliability^10^, and leading to blackouts that can ultimately leave populations without power precisely when cooling, and the electricity it relies on, are most needed.

Future cooling needs for adaptation to climate change can potentially exacerbate the difficulty and cost of mitigation, by increasing the demand for electric power, and in turn, the demand for energy carriers used for power generation. Integrated assessment model (IAM) simulations suggest that accommodating the climate-induced changes in energy demand^11^ will require substantial additional generation capacity and increased use of fossil fuels, leading to more CO$_{2}$ emissions and necessitating more stringent mitigation policies to achieve decarbonization^7^. The coarse spatial and temporal aggregation of IAM projections does not allow cooling adaptation’s role in expanding electricity use, or its subsequent effects on mitigation challenges, to be pinpointed. Instead, the growing availability of high-frequency (hourly or daily) electric load and weather provides an opportunity to address this disconnect through empirical modeling. Fine temporal scale co-variation between load and temperature can identify the impacts of transient extreme heat exposures, conditional on electricity consumers’ adjusting their utilization of stocks of energy-using durable goods that are fixed in the short run. This adaptation, along the so-called “intensive margin”^12^, has been inferred from comparatively short (< 10 y) data series^13–16^. The challenge is to empirically identify simultaneous adaptation along the “extensive margin”: consumers’ responses to average weather conditions experienced over many years that drive new technology adoption, and/or adjustment of stocks of appliances with varying energy efficiencies-direct observations of which are rare^17,18^. Here we estimate the increase in energy circa 2050 as a consequence of residential cooling adaptation, and draw implications for decarbonization in two very different regions, Europe and India, which together account for a quarter of the world’s population and 20% of global electricity consumption. We quantify the effects of mid-century climate change on daily peak electricity demand, accounting for endogenous increases in the prevalence of residential AC that amplify electricity consumption. Our results highlight that the benefit of reduced exposure to extreme heat comes at the cost of increased carbon dioxide (CO_2_) emissions and associated mitigation challenges in power systems that are not decarbonized. Specifically, we estimate the synergistic impact of long-run (extensive-margin) AC adoption and short-run (intensive-margin) AC utilization on the electricity demand response to daily maximum temperatures. Different from prior approaches that rely on realizations of weather that are contemporaneous with observed energy use^13,14,16^ or use dynamic proxies for the effect of accumulation of stocks of energy-using durables^18,19^, our analysis proceeds in four stages. First, we empirically model adjustment on the extensive margin, modeling the adoption of AC across regions and years in response to spatial and temporal differences in integrated heat exposure and income. Our second step is to empirically model the high-frequency intensive margin component of electricity demand, captured by the day-to-day co-variation between peak and total load and maximum daily temperature at different levels of regional AC prevalence. In the third step, we couple the intensive and extensive margin adaptation responses with mid-century projections of changes in daily maximum temperatures simulated by 29 global climate models (GCMs) to project the contribution of the extensive- and intensive-margin, as well as their joint amplifying effect on peak and total electricity consumption, at mid-century. Finally, we use projected changes in AC prevalence, electricity use and associated emissions to develop insights regarding the tradeoff between climate change mitigation and adaptation.

## Results

### Residential AC prevalence and its drivers

**Figure 1:**
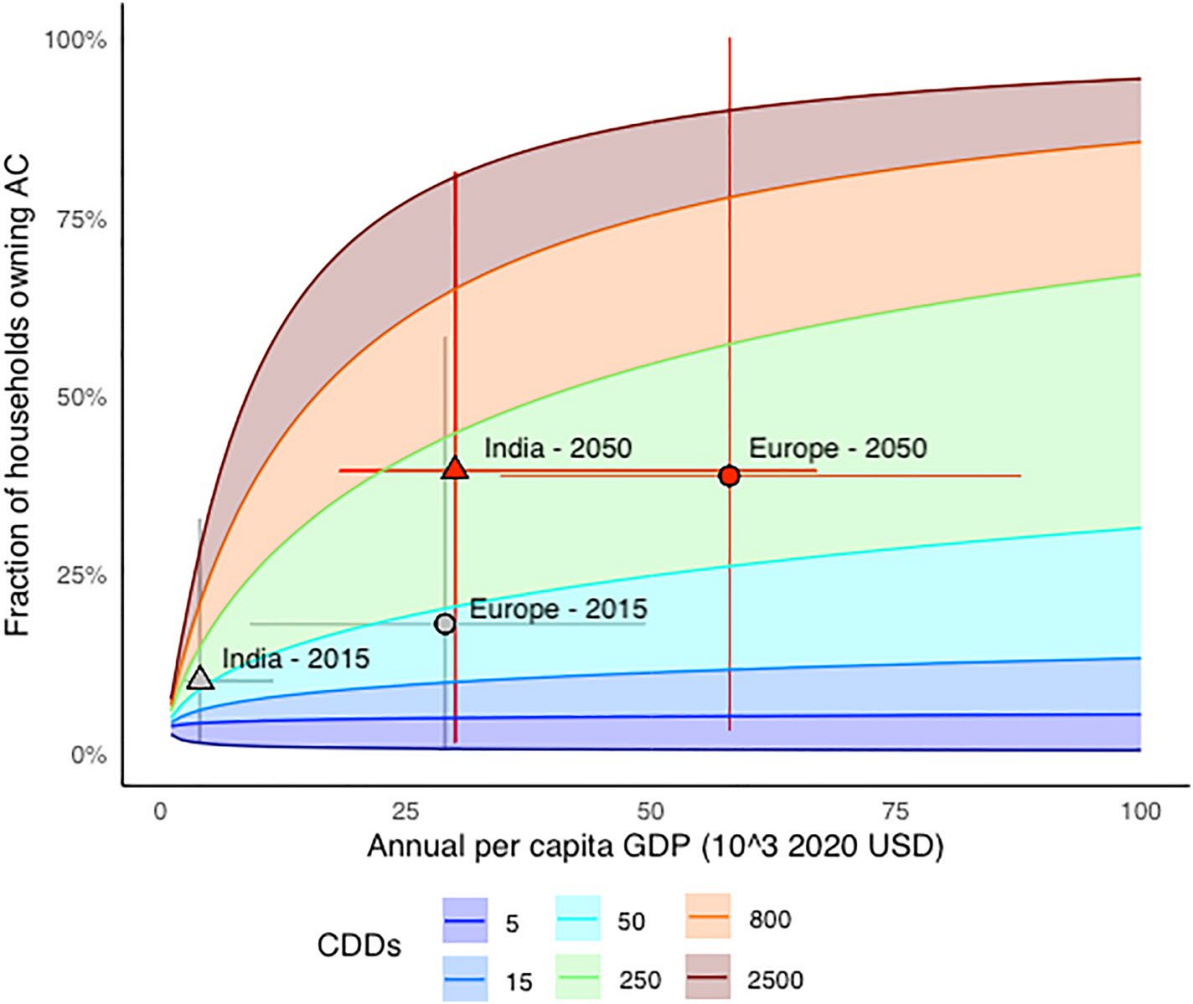
The combined effects of income and heat exposure on AC prevalence. Color ranges represent the income-AC curves at different levels of exposure to CDDs under the median urbanization level. Points represent the observed regional AC ownership rates, as well as the prediction at mid-century under RCP 8.5 and SSP 5. Regions’s vertical movements between zones with different colors indicate variations in AC prevalence due to an increase in the exposure to long-run CDDs, horizontal movements within zones of a given color indicate the effect of higher annual per capita income. Error bars indicate the 10th–90th quantile of per capita income (horizontal) and AC prevalence (vertical) across the States in the two regions in 2015 and circa 2050.

We empirically model the cross-jurisdictional, time-varying dynamics of central or room AC prevalence in the 47 sub-national administrative units, which we refer to as states (Members States in Europe and federal States in India) across two regions: India and Europe (see “Methods”). AC adoption responds non-linearly to per capita income and cooling degree days-the annual sum of daily average temperature exceedances above 24 °C threshold (CDD24, see “Methods”). AC prevalence increases strongly where the climate is warm (CDD24>250) and annual per capita income exceeds 20 k$, reaching rates as high as 50–70%. In areas with cool climates (CDD24<15), it saturates at 15–25%, irrespective of per capita income. Urbanization further amplifies prevalence, independently of income and temperature (see “Methods” and Supplementary Information, SI). Our empirical results suggest that Europe’s current per capita income is already high enough to support widespread adoption of AC, but moderate ambient temperatures have kept prevalence low. Conversely, India’s low per capita income constrains a household’s ability to acquire air conditioners, despite the high heat exposure. Thus, AC growth will respond more strongly to temperature in Europe’s richer temperate member states, and more strongly to income in India’s poorer hotter states. By coupling our adoption model with projections of CDD24s, income and population circa 2050, impacts of future temperature increases in Europe -causing AC prevalence to more than double from 19 to 41%—are inferred from a synthetic richer current India. The effects of future economic development in India—causing AC prevalence to increase fourfold from 10 to 40% (see Fig. 1 and SI)—are inferred from a synthetic hotter current Europe. The resulting convergence in AC prevalence does not close the current gap between developed and developing countries in the vulnerability to heat exposure: the 900 million Indian households that lack AC circa 2050 will be exposed to substantially higher temperatures than their European counterparts.

### Peak load temperature response conditional on AC

**Figure 2:**
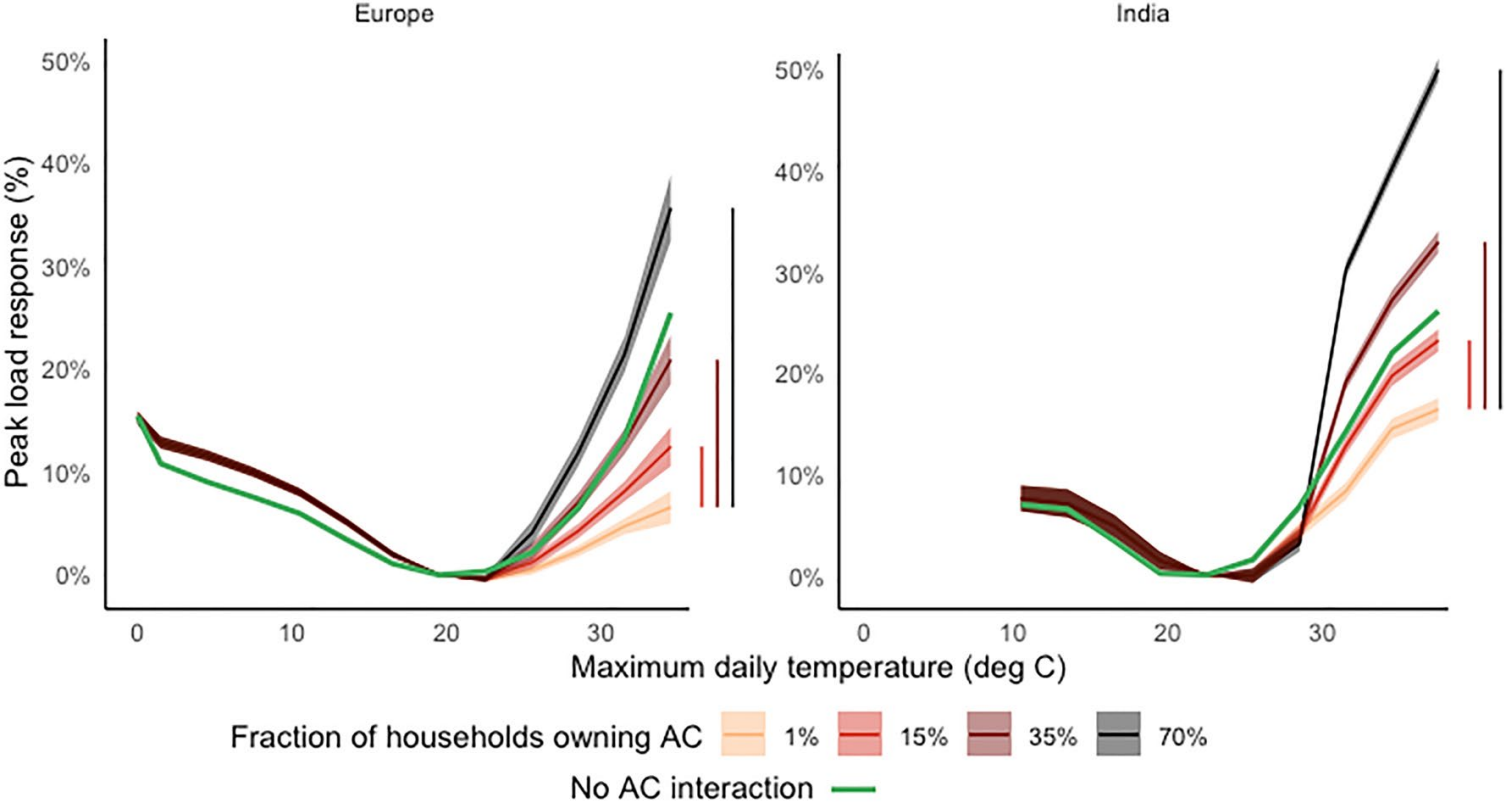
Response of per capita daily peak load to maximum daily temperatures. Shaded areas represent the 95% Confidence Interval (CI) of the empirically estimated response (see “Methods”). Vertical bars show the difference between the estimated response when AC $>0\%$ and when AC $\approx$0% for a $>33\,^\circ$C ($>36\,^\circ$C ) day in Europe (India), indicating the contribution of a given AC prevalence level once the effect of energy-intensive durable goods other than AC is filtered out.

Per capita daily peak and total load responses to maximum daily temperature (Figs. 2, S4) exhibit the U-shape previously found for mid-latitude locations [12, 13, 15, 19], in which the minimum of the functions corresponds to the non-weather sensitive per capita peak load (0.65 kWh in Europe, 0.12 kWh in India). The response to maximum temperatures, obtained without accounting for the AC prevalence modulation (the green curve in Fig. 2), rises from a + 10% increase at 24–27 $^\circ$C to a + 25% increase when temperature is above 33 $^\circ$C in both macro-regions. The benchmark response falls within the range of AC-dependent responses identified though the interaction effect. AC prevalence non-linearly amplifies the response of the peak load to temperatures above 24 $^\circ$C (Fig. 1 and “Methods”). The amplification for a $>33 \,^\circ$C day in a State with a 70% AC prevalence rate (i.e. above 95th percentile of the two regions’ distributions), such as Greece or Delhi is more than two times as large as the response under the mean AC prevalence in Europe (20%) and India (13%) (from +14% to +36% in Europe and from +21% to +49% in India). The amplification effect of AC is much larger in the $>33 \, ^\circ$C range than around 24–27 $^\circ$C, suggesting that the intensity of utilization of cooling equipment increases disproportionately with extreme high temperatures. Because of the two- to threefold increase in the mean AC prevalence level projected in the two regions circa 2050 (see Fig. 1), the expected growth in the cooling needs can potentially drive large amplifications in hourly peaks of future electricity consumption. Weather-driven peak load fluctuations that currently characterize high AC prevalence sub-regions will become widespread throughout Europe and India by mid-century. At negligible ($\approx$ 0%) rates of AC penetration, a $> 30\,^\circ$C day is associated with an average increase above the minimum of 6% in Europe, but 20% in India. This result suggests that India’s population, lacking access to AC, may rely heavily other electricity-using appliances (e.g., fans) as a substitute cooling technology to adapt to that country’s intense heat exposure, varying in the region from 143-1325 annual CDD24 (5–95% quantiles)^4^. Controlling for the effects of electricity-using appliances other than AC, peak load shocks for a given exposure to hot temperatures and AC ownership rate are very similar across the two marco-regions (vertical segments in Fig. 1). The responsiveness of the peak load to maximum temperatures begins to saturate only above 36 $^\circ$C (separate observations of the 33–36 $^\circ$C and $>36\,^\circ$C bins could be identified only for in India).

### Substantial amplification of peak and total load circa 2050

**Figure 3:**
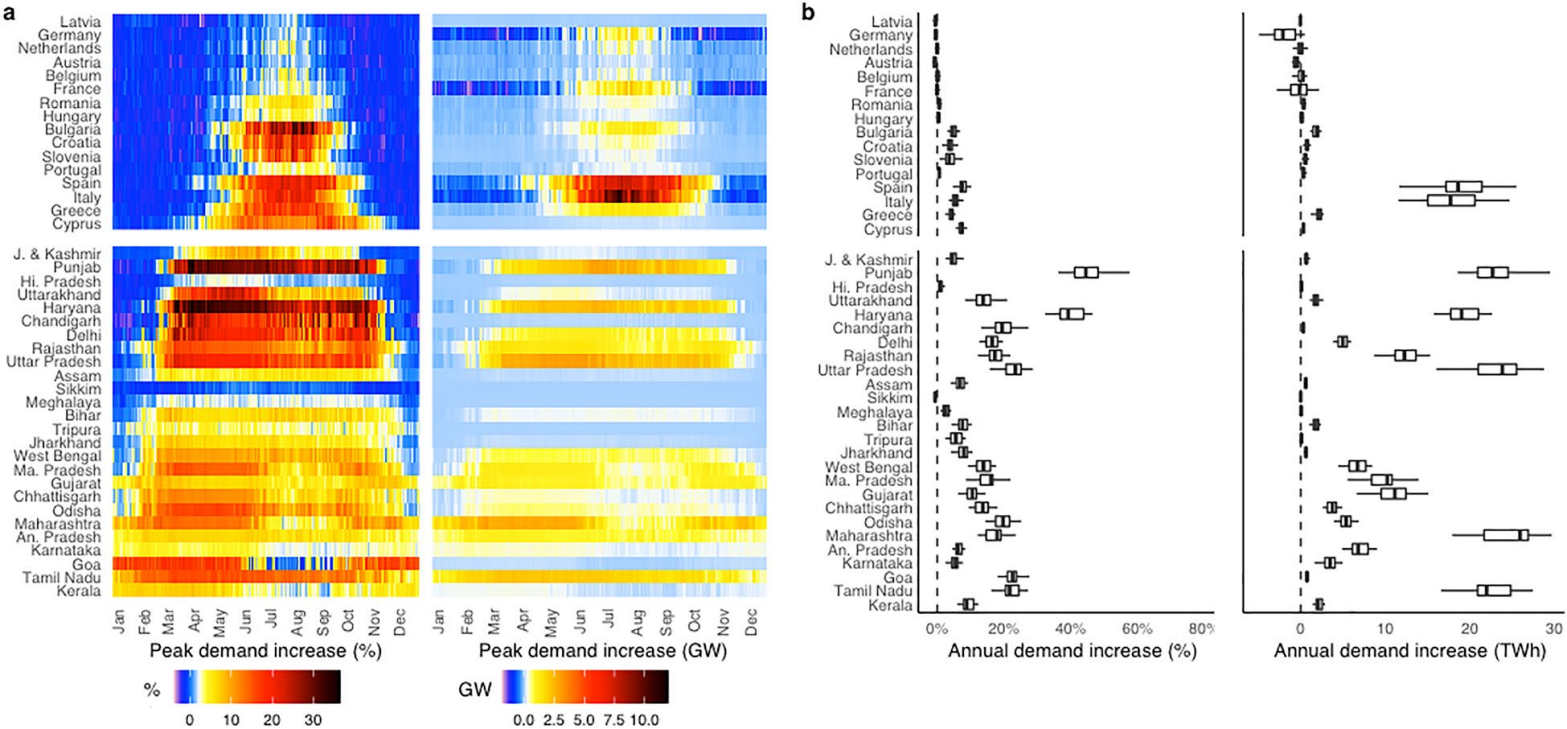
Amplification of peak and total load circa 2050. (**A**) Relative increase in the daily peak load, median of 29 GCMs (left) and absolute increase in the daily peak load, median of 29 GCMs (right) circa 2050. Historical daily peak load is computed as the daily average of 2010–2019. (**B**) Relative increase in the annual total load across 29 GCMs (left). Absolute increase in the annual total load across 29 GCMs (right).

At mid-century, hotter daily maximum temperatures in conjunction with higher AC prevalence synergistically increase the amplitude of daily peak and total load variations over the course of the year (see Fig. 3). European impacts exhibit a strong North-South gradient previously found by^13^. In Northern regions with mild summers, AC prevalence remains low, with higher warm season temperatures contributing to slight (5%) increases in summer peak demand that do not offset winter peak declines that accompany decreased heating requirements. Conversely, in Southern regions daily summer peak demands increase by 20–30%. Substantial additional peak generation capacity will be necessary to accommodate higher cooling demand in Southern Europe (Italy: 13 GW and Spain: 10 GW) and parts of India (Punjab, Uttar Pradesh and Maharashtra: 4 GW). The latitudinal gradient of impacts is weaker in India, where seasonal patterns are broadly similar across the majority of states. Except for the December–March dry season, fractional peak demand increases exceed those in Europe. North-Western India’s demand increases by more than 35%, due to the combination of substantial maximum daily temperature increases and high future AC prevalence induced by income growth (approaching 100% in Punjab, Haryana and Chandigarh, see SI). By contrast, low-latitude Indian states are characterized by a near-monotonic demand response, where the diurnal maximum temperature range (24–40 °C) corresponds to the portion of Fig. 2 to the right of the nadir, while temperate Indian states and European countries trace out the non-monotonic portion of response over a diurnal maximum temperature distribution with a lower support (0–33 °C). This result is further indication that climate change impacts on energy demand are unlikely to keep increasing all the way to the equator^16^. Total annual electricity demand in Europe increases by roughly 34 TWh, or 2% of today’s consumption, with Southern countries’ additional 40 TWh of demand offset by mild decreases in consumption in Northern countries. Conversely, annual electricity demand in India grows by as much as 168 TWh, or 15% of today’s consumption. The main features of our results are not meaningfully affected by inter-model uncertainty in GCM projections (Fig. 3b).

### Extensive versus intensive margin adaption

**Figure 4:**
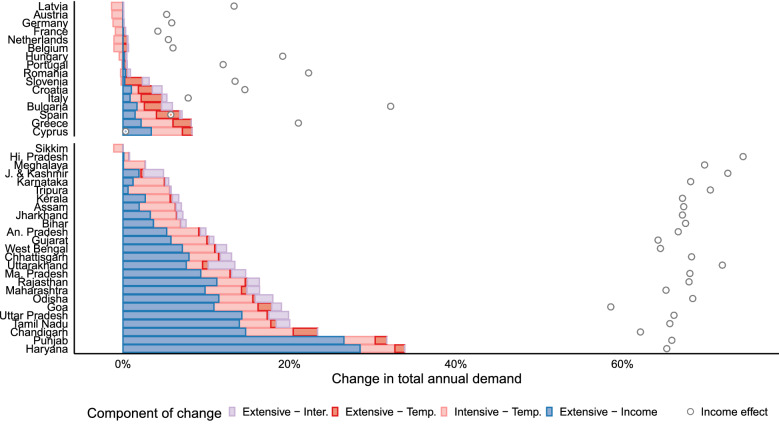
Decomposition of changes in total annual electricity demand in 2050 relative to present. Demand amplification at the climate-driven intensive margin and the climate and income-driven extensive margin components (coloured bars)—median of 29 GCMs. The circles represent the additional total annual load increase due to the change in future per capita income, and are hence unrelated to cooling (income effect).

Higher daily maximum temperatures circa 2050 increase total annual electric load along the intensive margin, i.e. adjustments in households’ and firms’ utilization of their current appliance stocks. In Europe, this effect is positive and large in Southern countries with high AC prevalence that experience hotter summers (Cyprus, Greece, Spain), and negative and small in Northern countries that experience milder winters (Fig. 4). India experiences a positive intensive margin amplification that is more geographically uniform, largely due to the monotonic demand response exhibited by states with tropical climates. Extensive margin impacts arise from adjustments in residential appliance stocks driven by long-run increases in CDDs and per capita income, holding constant patterns of AC utilization based on the current temperature distribution. Concomitant increases in total annual load are very large in India, where income-driven increases in AC ownership account for more than two-thirds of the total shift in the demand of most states. This effect is exacerbated by mid-century increases long-run heat exposure, but the extent of amplification is modest, comparable in size to the extensive-margin impacts of both heat and income in Southern Europe. Figure 4 indicates that the largest single component of change is the intensive margin effect of income on appliance utilization, which corresponds to an upward shift in the nadir of Fig. 1’s temperature-demand responses. Projected per capita income growth to 2050 under the SSP5 scenario amplifies total annual load by 60–70% across India, 25–35% in Bulgaria, Slovenia, Hungary and Greece, but less than 15% in the rest of Europe (Table 1).

**Table 1:** Decomposition of annual electricity demand increase.

	A. Decomposition components	
	Europe	India
	TWh (%)	TWh (%)
Intensive: income	156.5 (7.5)	718.2 (66.5)
Intensive: temperature	− 0.5 ($\approx$ 0)	40.2 (3.7)
Extensive: income	8.8 (0.4)	110.4 (10.2)
Extensive: temperature	20.5 (1.0)	4.5 (0.5)
Interactions	4.3 (0.2)	12.8 (1.2)
Intensive: total	− 0.5 ($\approx$ 0)	40.2 (3.7)
Extensive: total	33.6 (1.6)	127.7 (11.9)

### Mitigation-adaptation tradeoffs

**Figure 5:**
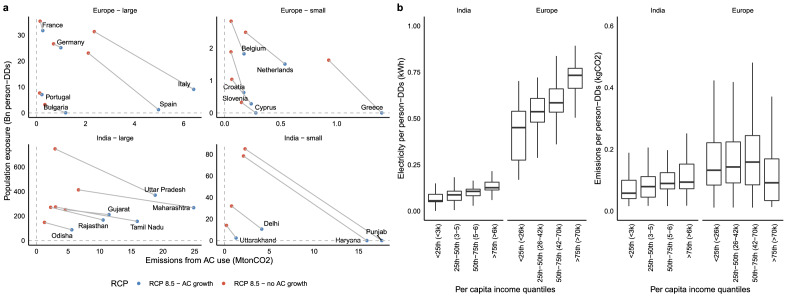
Annual exposed population and carbon emissions under alternative AC prevalence scenarios circa 2050. (**a**) State-level variations induced by climate change circa 2050 in the annual CO_2_ emissions from power generation and annual count of person-degree days exposed to daily maximum temperatures above 24 °C, respectively with (blue) and without (red) the projected growth in AC prevalence. States are grouped into different panels depending on the size of the population exposed, with “small” countries having annual exposed person-DDs below 3 billion in Europe and below 100 billion in India. The states with the smallest population counts have been removed to avoid clutter, the figure showing all states is shown in the Supplementary Results. (**b**) Variation in the electricity demand and carbon emissions from a unitary reduction in person-degree day exposure by each grid-cell, across income groups, circa 2050 under the RCP 5-8.5.

Increasing AC prevalence yields health benefits of reduced population exposures to heat stress, and environmental costs of additional emissions from generating electricity used for cooling. Our metrics are, for the former, the count of person-degree days (PDDs), i.e. the number of people without AC in their homes who are exposed to maximum daily temperatures above 24 °C, and for the latter, the annual CO_2_ emissions from the additional electricity generation associated with AC use, assuming the current power generation mix (see “Methods”). Figure 5a illustrates these metrics circa 2050 for the intensive margin case of increased utilization of today’s appliance stock with holding AC prevalence constant, and the extensive margin case where AC prevalence adjusts according to Fig. 1. In Indian states and Southern European countries, heat exposure reductions and CO_2_ emissions increases are large, indicating substantial benefits at the cost of exacerbated mitigation challenges. Figure 5b summarizes the heterogeneity in this tradeoff, in terms of gridcell level variation in the increase in electricity demand and CO_2_ emissions from a one PDD reduction in heat exposure. The marginal increases in demand are four times as large in Europe as they are in India, while marginal increases in emissions are almost twice as large in Europe, reflecting India’s more carbon-intensive power generation mix. Within the two regions, marginal energy use and emissions generally increase with the level of income: states with per capita incomes in the highest quartile see 50–75% more severe tradeoffs compared to states with per capita incomes in the lowest quartile. This trend is reversed only for the richest European countries with large shares of hydropower generation. Overall, growth in AC prevalence to mid-century is responsible for increasing annual emissions by 7–17 MTCO2 in Europe and 38-160 MTCO2 in India, while reducing daily heat exposures from 430 million to 265 million PDDs in Europe, and from 11.1 billion to 7.3 billion PDDs in India. These additional emissions are substantial, corresponding to 2% and 15% of current power sector emissions in Europe and India, respectively. Moreover, compared to Europe, India will see a tenfold larger population without AC exposed to $> 24\,^\circ$C maximum temperatures (Fig. S6 and Table S2).

## Discussion

Over the coming decades, humankind will need to simultaneously adapt to hotter ambient temperatures and avert further warming by mitigating emissions of heat-trapping gases^20^. We have highlighted the tension between these actions, quantifying mitigation challenges arising from the energy demand consequences of temperature-driven adoption of AC. Fortunately, such mitigation-adaptation tradeoffs can be moderated, if not avoided entirely, through various means. These include increasing electricity supply while reducing the emission intensity of generation-or the electricity intensity of economic activity more broadly, increasing the energy efficiency of cooling appliances in general and AC specifically, and incentivizing behavioral changes in cooling. Additional non-dispatchable renewable or dispatchable low-carbon generation allows the electricity demand increases from expanded cooling to be accommodated without increasing emissions. The latter could be achieved through reductions in electricity’s CO_2_ intensity that are modest in Europe-from 270 to 265 gCO_2_/kWh, but substantial in India-from 775 to 700 CO_2_/kWh, corresponding to displacement of 147 TWh of coal generation annually, or retirement of 17 GW of capacity, around 7% of India’s current coal fleet^21^. Where CO_2_ emissions are regulated, such reductions can be achieved though more stringent abatement targets or higher carbon prices. The relationship between carbon prices and regional power sector CO_2_ intensities in^7^ suggests that the necessary price increases would be on the order of 5–30%. Projected increases in residential electricity demand could also be accommodated via demand-side measures that reduce power consumption across a range of economic activities. Extrapolating historical trends, by 2050 India’s electricity intensity of GDP is comparable to that of Europe today, while Europe’s is some 30% lower-within the range simulated by^7^. As before, the additional aggregate intensity declines necessary to offset the consequences of higher residential AC prevalence are small in Europe (from 0.102 to 0.10 kWh/$) but substantial in India (0.149 to 0.135 kWh$). Focusing on AC specifically, end-use efficiency improvements could facilitate reductions in heat exposure with smaller increases in electricity consumption. Improving AC units’ seasonal energy efficiency ratios (SEERs) from their current region-specific average levels to their best available levels^8^ could moderate annual electricity consumption increases by 50% (17 TWh) in Europe and 40% (109 TWh) in India (SI). Inducing changes in households’ cooling behavior can also moderate electricity consumption increases. One such response is cooling technology substitution. While ventilation is less effective than AC for reducing thermal discomfort, it uses much less energy. Recent findings suggest that coupling AC with fans might allow comfortable indoor temperatures to be maintained with up to 76% less additional electricity^22^. Using the relationship between air speed and air temperature from^23^, simple calculations suggest that, even when the additional electricity consumption from running fans is accounted for, operating an AC unit at a higher temperature threshold could lower household annual electricity consumption by 40–60% in Europe and 50–60% in India, depending on the temporal pattern of ventilation (SI). Additional responses, whose attractiveness and efficacy are difficult to quantify, involve affected populations shifting activities in time and space to avoid heat exposure^24^. Outdoor activities could be shifted to cooler hours of the day, while hot hours might be spent in public or private air conditioned environments outside the home (e.g., malls, offices, or even vehicles). Our residential AC-based electricity demand amplification estimates do not account for potential future adjustments in commercial cooling capacity, utilization or energy consumption. If the latter are subject to economies of scale, their use as substitutes for residential AC could make mitigation-adaptation tradeoffs less severe. Our decomposition results suggest that the relative attractiveness of the above options will vary within and across regions. In northern Europe, where amplification of heat exposure and electricity demand are both small, adaptation could prioritize low-cost activity shifting or ventilation. Southern Europe’s larger amplification effects, with similarly-sized temperature-driven intensive margin and income-driven extensive-margin components, suggest behavior-based policies to incentivize ventilation during the shoulder seasons, augmented by appliance efficiency standards and subsidization of energy efficient AC. In India, where income-driven extensive-margin adjustments are the major driver of amplification and current market average SEER values are low, appliance efficiency standards coupled with subsidization of energy efficient AC could be prioritized. Assessment of the regionally cost-effective mix of such strategies is left to future research. Planned adaptation strategies, while not specifically designed to address the mitigation-adaptation tradeoff, may nonetheless help to moderate it. Passive cooling, reflective roofs and urban greening^25^, as well as more energy efficient buildings, could significantly reduce the energy requirements needed to adapt to extreme temperatures^26,27^, while smart grid and computerized building energy management systems could shift and/or shave power demand peaks at times of maximum cooling load^28^. Even so, we emphasize that, under the assumptions of the SSP5 and SSP2 future scenarios, AC ownership is likely to remain unaffordable for sizeable populations. Around2050, 60 million European and nearly 640 million Indian residents will be exposed to high temperature extremes without AC at home. How to provide cooling for populations that are low-income, vulnerable to heat, and lack the capacity to adapt, is a question that is ripe for investigation. Methodologically, our empirical framework facilitates decomposition of extensive- and intensive-margin energy demand adaptations to rising ambient temperatures, allowing us to compare the contributions of warming and economic development to future peak and daily electricity demand shifts. Prior attempts to quantify extensive-margin amplification have exploited annual cross-sectional variation between households^17,18^. Here we rely on a cross-jurisdictional panel dataset tracking AC prevalence rates over two decades, and exploit the economic and climatic differences between and within Europe and India for the identification of the adoption function. Our results of mid-century Indian average adoption rates are close to the lower range of the estimates by^4^, that span from 49 to 69%. Similarly to^4^, we find a high sensitivity of future AC adoption rates in India across socio-economic scenarios (see Supplementary Information). Differences with respect to^4^ may be attributed to our inability to control for education and housing conditions, which can only be adequately measured at the household level. Furthermore, while we control for urbanization effects, urbanization is taken as given in our projections. Accounting for the increase in urbanization across SSPs would hence lead to higher adoption rates and further amplify the extensive margin-adjustments presented in this study. Our intensive-margin projections of peak electricity demand amplification in Europe are in line with^13^, while impacts that include the effects of extensive-margin AC adjustments are 2 to 3 times larger in southern Europe. Compared to impact projections based solely on responses to temperature extremes^11^, our non-linear demand response function (Fig. 2) captures the effects of climate change-driven shifts in the temperature distribution with greater fidelity, and yields substantially smaller mid-century electricity demand amplification. Exploiting the high-frequency weather-load co-variation and accounting for the direct modulation effects of AC uncovers how middle-income developing economies such as India exhibit similar responses to high-income regions such as Europe, an effect that calls into question previous studies estimating a flat load-weather response pattern in low- and middle-income regions^6^. Our results underscore the importance of modeling adaptive behaviors as a response to both socio-economic and climatic changes, pointing to the need of teasing out extensive margin adjustments in frameworks relying on more aggregate cross-country data. We close with a discussion of caveats. First, our simplifying assumption that AC is the only factor mitigating heat exposure in households when we estimate the number of people exposed to extreme temperatures. This approximation can suffer from bi-directional biases: upward, due to our inability to observe and model the cooling benefits of fans or building insulation, and downward, due to the challenge of accounting for outdoor exposures by members of households that do own AC. Second, lack of sector-specific high-frequency electricity demand data and AC ownership statistics prevent us from identifying adjustments in commercial AC adoption and their implications for aggregate electricity demand. It is therefore likely that our findings understate the effects of adaptation at the extensive margin, especially given the sensitivity of commercial sector electricity demand to high temperatures and economic growth found by^11,19^. Finally, we have examined only the impacts of changing temperatures, even though a key aspect of the thermal comfort benefit of AC is dehumidification. We leave to future research empirical investigation of the electricity demand effects of humidity, particularly its role in maintaining thermal comfort in tropical regions. Although prior studies have identified only minor influences on households’ responses^4,29^, there is the potential for larger impacts on peak demand.

## Methods

### Approach

As summarized in the introduction, our analysis proceeds in four phases.

- I. Empirical modeling of the low-frequency extensive margin of AC prevalence across regions and years in response to spatial and temporal differences in heat exposure and income.
- II. Empirical modeling of the high-frequency intensive margin of electricity demand, captured by the day-to-day co-variation between peak and total load and maximum daily temperature, conditional on AC prevalence.
- III. Projection of electricity consumers’ adjustments along the extensive and intensive margins, and the total impact on electric power demand, circa 2050, by coupling the fitted empirical models from I and II with GCM-simulated temperatures and scenarios of future GDP.
- IV. Computation of the associated mid-century increases in CO_2_ emissions and comparison the avoided exposure of population to thermal discomfort thanks to the increase in AC prevalence on the basis of projections (III) and by using decomposition analysis to elucidate the relative importance of intensive and extensive margin cooling adaptation.

### Data

We assemble two longitudinal datasets. For phase I of our analysis, we use a dataset of annual rates of AC ownership covering 17 European countries over the period 1990–2019^30^ and 30 Indian states over the period 2013–2019^31^. For phase II we use a dataset of daily peak and total electric load covering 16 European countries over the period 2015–2019^32^ and 28 Indian states over the period 2013–2019^33^. Each dataset is matched to population-weighted temperature exposures, computed from ERA5 0.25 hourly 2m temperature series^34^, as well as annual real per capita GDP in 2015 US dollars^35,36^. The first low-frequency dataset is used to empirically model the drivers of AC adoption across an inter-regional gradient of income and climatically-determined heat exposure. To match the time-step of the outcome variable, diurnal average temperatures are computed and aggregated over the course of each year to construct population-weighted CDD24s as a measure of integrated heat exposure. We use the second set of high-frequency data to analyze the contemporaneous effect of heat on the per-capita demand for electricity, conditional on the prevalence of AC. To that end, daily peak and average electric load are matched to diurnal maximum temperatures and annually-varying GDP and AC prevalence (Supplementary Methods and Table S1).

Phase III employs future estimates of gridded global population from^37^ and GDP from^38^, developed in accordance with the shared socioeconomic pathway (SSP) scenarios. Shifts in CDDs and daily maximum temperatures from current to mid-century climates are estimated using the outputs of 29 global climate models (GCMs) participating in the Coupled Model Intercomparison Project, Phase VI (CMIP6)^39^. Specifically, we use GCM-simulated daily temperature fields for moderate (SSP245) and vigorous (SSP585) warming scenarios that are bias corrected and downscaled to a 0.25 grid, from the from the NASA NEX-GDDP-CMIP6 dataset^40–42^ (further details are provided in the SI). In phase IV, we estimate future CO_2_ emissions from electricity generation using technology-specific power generation data recorded on a daily time-step for European countries^32^ and on a monthly time-step for India’s five electricity dispatch regions^36^, over the period 2017-2019. We couple power generation statistics with carbon intensity associated with the operation of power plants available at the country level for Europe^43^ and at the national level for India^44^.

### Empirical analyses

#### The extensive margin: modeling AC prevalence

In each location (*i*) and year (*t*), the probability of AC ownership is approximated by the share of households with AC (*s*), which we model as a function of the 10-year moving average CDD24s (${\mathscr {C}}$), the logarithm of the 10-year moving average annual per capita income (*y*) and the logarithm of the 10-year moving average annual urbanization rate (*u*). Our dependent variable is continuous on [0,1]. Our empirical specification is a pooled cross section-time series regression with a logit link function, $\Lambda$^45^:

1 $$\begin{aligned} \Lambda (s_{i,t})&= \log \left( \frac{s_{i,t}}{1-s_{i,t}} \right) = {\varvec{Z}}\varvec{\alpha } \nonumber \\&= \alpha _i^0 + \alpha ^Y y_{i,t} + \alpha ^C {\mathscr {C}}_{i,t} + \alpha ^{YC} (y_{i,t} \cdot {\mathscr {C}}_{i,t}) + \alpha ^U u_{i,t} \end{aligned}$$

with location fixed effects $\alpha ^0$, and estimated parameters $\alpha ^Y$ and $\alpha ^C$ that capture the direct effects of income and heat exposure, and $\alpha ^{YC}$ that captures their interaction. The functional form yields nonlinear effects of the linear predictors, governed by the logistic transformation:

2 $$\begin{aligned} {\widehat{s}} = \Lambda ^{-1}\left( {\varvec{Z}}\widehat{\varvec{\alpha }}\right) = \frac{\exp \left( {\varvec{Z}}\widehat{\varvec{\alpha }}\right) }{1 + \exp \left( {\varvec{Z}}\widehat{\varvec{\alpha }}\right) } \end{aligned}$$

#### The intensive margin: modeling peak and total load conditional on AC

We characterize the responses of European and Indian peak and total electricity demand to high temperature exposure on a daily time step. We bin population-weighted diurnal maximum temperatures into *k* intervals of 3 °C width, $B_k=[{\underline{T}}_k, {\overline{T}}_k)$, and construct a *k*-vector of indicators that track whether each day’s maximum temperature falls within a given interval:

$$\begin{aligned} {\mathscr {T}}_k = 1 \cdot \lbrace T \in B_k \rbrace + 0 \cdot \lbrace \text {Otherwise}\rbrace \end{aligned}$$

Bins are differentiated by region to account for the large climatic differences—Europe: $\{<0, 0-3,..., 30-33,>33\}$, India: $\{<12, 12-15, ..., 33-36,>36\}$. The resulting indicator variables are employed as high-frequency covariates in regionally-stratified linear fixed effects models of per capita daily electric load, $q_v$, where the subscript $v = \lbrace \text {Peak, Total} \rbrace$ indexes peak or total demand^13,14,19,46^. Suppressing location and time subscripts, our empirical specifications are:

3 $$\begin{aligned} {\mathbb {E}} [\ln q_v]&= \textstyle \sum _k \beta _{k,v}^T {\mathscr {T}}_k + \beta _v^Y y + \text {controls} \end{aligned}$$

4 $$\begin{aligned} {\mathbb {E}} [\ln q_v]&= \textstyle \sum _k \beta _{k,v}^T {\mathscr {T}}_k + \sum _k \beta _{k,v}^{TAC} \left( {\mathscr {T}}_k \cdot s \right) + \beta _v^Y y + \text {controls} \end{aligned}$$

where controls include state or country fixed effects that absorb variation associated with unobserved temporally-invariant confounders, and day-of-week, season and year fixed effects that control for idiosyncratic time-varying influences that are unrelated to temperature. Both models are estimated by OLS, with standard errors robust to heteroscedasticity and serial correlation and clustered at the state level (see SI for additional details).

Specification (3) follows the empirical approach in prior literature, in which the parameters are identified based on contemporaneous co-variation between electricity demand and realizations of weather^13,14^. In particular, $\varvec{\beta }^T$ is identified off the deviations of observed daily load and binned temperature exposures from their local average values—shocks which are informative of the average short-run response across locations. The elements of $\varvec{\beta }^T$ trace out the intensive margin response of energy demand to temperature, not accounting for consumers’ adjustments of stocks of energy-using durables. The potential amplification of demand due to latter^2,4^ is explicitly captured in our preferred specification, (4), by the vector of interaction coefficients, $\varvec{\beta }^{TAC}$. The fitted coefficient vectors $\widehat{\varvec{\beta }}^T$ and $\widehat{\varvec{\beta }}^{TAC}$ provide flexible piece-wise linear spline representations of macro-regions’ distinct nonlinear temperature response functions (Fig. 2 for the peak load and Supplementary Fig. 3 for the total daily load).

#### Impact projections

The second stage of our analysis combines the estimated parameters $\widehat{\varvec{\alpha }}$ and $\widehat{\varvec{\beta }}$ with climate change projections to estimate the impacts of mid-century temperature increases on peak and total electricity demands, conditional on the future level of AC ownership. We use representative 5-year periods from the current (2010–2014) and future (2055–2059) epochs, indexed by by the superscripts $e = \lbrace C, F \rbrace$. Within each epoch we compute at the grid cell level, for each year, the 10-year moving average CDDs, $\overline{{\mathscr {C}}}^e$, and, for each day, the contemporaneous maximum temperature interval, $\overline{{\mathscr {T}}}_k^e$. Following^47^, climate change-driven temperature shifts were estimated by calculating the differences between simulated 10y average annual CDDs and daily maximum temperatures over the historical and future epochs, and adding these “deltas” to the corresponding series of historical observations recorded by ERA5. The resulting series for the current and future epochs, $\widetilde{{\mathscr {C}}}^C$, $\widetilde{{\mathscr {T}}}^C_k$ and $\widetilde{{\mathscr {C}}}^F$, $\widetilde{{\mathscr {T}}}^F_k$, are then used to project future AC prevalence in conjunction with (2), and the concomitant impact on daily peak and total per capita load in conjunction with (4). Let ${\overline{s}}^C$ denote the observed baseline AC prevalence, and

5 $$\begin{aligned} {\widetilde{s}}^C&= \Lambda ^{-1} \left[ {\widehat{\alpha }}^0 + {\widehat{\alpha }}^Y {\widetilde{y}}^C + {\widehat{\alpha }}^C \widetilde{{\mathscr {C}}}^C + {\widehat{\alpha }}^{YC} ({\widetilde{y}}^C \cdot \widetilde{{\mathscr {C}}}^C) + {\widehat{\alpha }}^U {\overline{u}} \right] \end{aligned}$$

6 $$\begin{aligned} \text {and} {\widetilde{q}}^C_v&= \exp \left[ \textstyle \sum _k {\widehat{\beta }}_{k,v}^T \widetilde{{\mathscr {T}}}_k^C + \sum _k {\widehat{\beta }}_{k,v}^{TAC} \left( \widetilde{{\mathscr {T}}}_k^C \cdot {\widetilde{s}}^C \right) + {\widehat{\beta }}_v^Y {\widetilde{y}}^C \right] \end{aligned}$$

denote predicted AC prevalence in response to historical cooling degree days and income, and predicted peak and total electricity demand in response to historical temperature and income, respectively. Projections of AC prevalence and electricity demand amplification are given by:

7 $$\begin{aligned} {\widetilde{s}}[{\widetilde{y}}^e, \widetilde{{\mathscr {C}}}^e] = \Lambda ^{-1} \left[ {\widehat{\alpha }}^0 + {\widehat{\alpha }}^Y {\widetilde{y}}^e + {\widehat{\alpha }}^C \widetilde{{\mathscr {C}}}^e + {\widehat{\alpha }}^{YC} ({\widetilde{y}}^e \cdot \widetilde{{\mathscr {C}}}^e) + {\widehat{\alpha }}^U {\overline{u}} \right] \cdot \frac{{\overline{s}}^C}{{\widetilde{s}}^C} \end{aligned}$$

8 $$\begin{aligned} \text {and} \psi _v \left[ {\widetilde{s}}[{\widetilde{y}}^e, \widetilde{{\mathscr {C}}}^e], {\widetilde{y}}^e, \widetilde{{\mathscr {T}}}^e \right] = \exp \left[ \textstyle \sum _k {\widehat{\beta }}_{k,v}^T \widetilde{{\mathscr {T}}}_k^e + \sum _k {\widehat{\beta }}_{k,v}^{TAC} \left( \widetilde{{\mathscr {T}}}_k^e \cdot {\widetilde{s}}[{\widetilde{y}}^e, \widetilde{{\mathscr {C}}}^e] \right) + \textstyle {\widehat{\beta }}_v^Y {\widetilde{y}}^e \right] / {\widetilde{q}}^C_v \end{aligned}$$

(7) and (8) are computed using each GCM’s simulated output at the grid cell level (*g*) for the 5y epoch and constitutent days (*d*), respectively. We leverage our observations of historical average per capita demand for *i* European countries and Indian states to aggregate the shocks using future (SSP2 and SSP5) gridded population, ${\widetilde{n}}^F$, as follows:

9 $$\begin{aligned} \Psi _{v,i} = \frac{\sum _{g(i)} \sum _d \psi _{v,d} [{\widetilde{y}}^F, \widetilde{{\mathscr {C}}}^F] \cdot {\overline{q}}_{v,i,d} \cdot {\widetilde{n}}_g^F}{\sum _{g(i)} \sum _d {\overline{q}}_{v,i,d} \cdot {\widetilde{n}}_g^F} \end{aligned}$$

Analysis of CMIP6 GCM outputs has shown that relying on multi-model ensemble medians may lead to higher projections of warming than the Intergovernmental Panel on Climate Change Sixth Assessment Report (AR6) assessed-warming averages^48^. In our sensitivity analysis we rely on the recent classification proposed by^48^, that identifies those GCMs that provide reasonable projections of warming consistent with the AR6, and those characterized by high-sensitivity, resulting in “too hot” projections. While we find only one clear outlier on the high end, we also identify a 10–18% difference between the impacts relying on the ensemble medians of the “consistent” vs “hot” model groups, depending on the region (see Supplementary Results).

#### Insights: impact decomposition, electric power emissions and population exposure to heat.

We decompose the amplification in electricity demand into the fractional effect of each intensive- and extensive-margin driver: the climate-induced intensive-margin adjustments that result from utilization of the historical endowment of appliances under a changed climate (10a); the climate-induced extensive-margin adjustments that result from the utilization of new AC endowments as a response to a changed climate (10b); the income-induced adjustments of temperature-independent load (i.e. the nadir of the load-temperature response function) due to per capita income growth (10c); the income-induced extensive-margin adjustments that result from the utilization of new AC endowments as a response to future per capita income (10d); the interaction effects due to the non-linearity of the concurrent effects of climate and income at the intensive- and extensive-margins (10e).

10a $$\begin{aligned} \psi _v^{TI}= & {} \psi _v \left[ {\widetilde{s}}[{\widetilde{y}}^C, \widetilde{{\mathscr {C}}}^C], {\widetilde{y}}^C, \widetilde{{\mathscr {T}}}^F \right] \end{aligned}$$

10b $$\begin{aligned} \psi _v^{TE}= & {} \psi _v \left[ {\widetilde{s}}[{\widetilde{y}}^C, \widetilde{{\mathscr {C}}}^F], {\widetilde{y}}^C, \widetilde{{\mathscr {T}}}^C \right] \end{aligned}$$

10c $$\begin{aligned} \psi _v^{YI}= & {} \psi _v \left[ {\widetilde{s}}[{\widetilde{y}}^C, \widetilde{{\mathscr {C}}}^C], {\widetilde{y}}^F, \widetilde{{\mathscr {T}}}^C \right] \end{aligned}$$

10d $$\begin{aligned} \psi _v^{YE}= & {} \psi _v \left[ {\widetilde{s}}[{\widetilde{y}}^F, \widetilde{{\mathscr {C}}}^C], {\widetilde{y}}^C, \widetilde{{\mathscr {T}}}^C \right] \end{aligned}$$

10e $$\begin{aligned} \psi _v^{Int}= & {} \psi _v - (\psi _v^{TI} + \psi _v^{TE} + \psi _v^{YI} + \psi _v^{YE} ) \end{aligned}$$

We compute CO$_2$ emissions associated to the additional power generation due to climate change circa 2050 assuming that power system carbon intensities correspond to the level observed in Europe and India in the three years from 2017 to 2019 (*n*) (see Supplementary Table 2 in the Supplementary Information). We derive regionally and seasonally-varying CO$_2$ emission intensities of the power system (${\mathscr {P}}_{g,i}$) weighting the technology-specific emissions intensities ($\rho _{i,f}$) by the historical regional- and seasonal- specific share of each technology (*f*) in the power generation mix ($\omega _{i,g,f}$), based on observed power generation (${\mathscr {G}}_{n,i,g,f}$).

11a $$\begin{aligned} \omega _{g,i,f}&= \frac{1}{n} \textstyle \sum ^{n}_{i=1} \left\{ {\mathscr {G}}_{n,g,i,f} / \sum _{f}{\mathscr {G}}_{n,g,i,f} \right\} \end{aligned}$$

11b $$\begin{aligned} {\mathscr {P}}_{g,i}&= \sum _{f} \rho _{i,f} \omega _{g,i,f} \end{aligned}$$

Where the subscript *i* indexes alternatively the states or the dispatch regions, and the subscript *g* indexes alternatively the day or month of the year, for Europe and India respectively. Average emission intensities (${\mathscr {P}}_{i,g}$) as well as the average regional share of each technology in the generation mix are shown in the Supplementary Results.

We compute the count of person-degree days exposed (PDDs), i.e. the number of people without AC in their homes who are exposed to maximum temperatures above 24 °C ($\Gamma _{i}$), as follows:

12 $$\begin{aligned} \Gamma _{i} = \sum _{g(i)} {\mathscr {C}}_{g} \cdot Pop_{g} \cdot (1-{\widetilde{s}}_{i}) \end{aligned}$$

Where the subscript *i* indexes the state, *g* the gridcell, and ${\mathscr {C}}_{g}$, $Pop_{g}$ and ${\widetilde{s}}_{i}$ are respectively the number of CDDs, the total population and the state-level AC prevalence projected in 2050.

## Supplementary Information

Supplementary Information.
