# Supplementary material for: Air-conditioning adoption and electricity demand highlight climate change mitigation–adaptation tradeoffs
Source: Sci Rep. 2023 Mar 17;13:4413. doi: 10.1038/s41598-023-31469-z (PMC10023814; doi:10.1038/s41598-023-31469-z)
Supplement: Supplementary file 1 — Supplementary Information. [file 41598_2023_31469_MOESM1_ESM.pdf]

Supplementary Information to: Air-conditioning adoption and electricity demand highlight climate change mitigation-adaptation tradeoffs

Francesco Pietro Colelli*[1,2], Ian Sue Wing[3], Enrica De Cian[1,2]

[1] Department of Economics, Ca' Foscari University of Venice, 30121 Venice, Italy

[2] Fondazione Centro Euro-Mediterraneo sui Cambiamenti Climatici (CMCC), 30175 Venice, Italy

[3] Boston University, Dept. of Earth & Environment, 685 Commonwealth Ave., Boston Massachusetts 02215, USA

1 Supplementary Methods

1.1 Data

Table 1: Data sources

Variable	Region	Source
AC ownership	Europe	ENERDATA-ODYSSEE MURE [11]
AC ownership	India	CMIE [7]
Electricity demand	Europe	ENTSO-E[3]
Electricity demand	India	CEA [2]
GDP and Population	Europe	Eurostat [4]
GDP and Population	India	Reserve Bank of India [10]
Downscaled population by SSP	-	Olen et al., 2022 [12]
Downscaled GDP by SSP	-	Murakami et al., 2021 [9]
Historical daily temperatures	-	ERA-5 Land [6]
Projected daily temperatures	-	NASA NEX-GDDP-CMIP [16, 15, 17]

Daily peak and total electric load are defined as the sum of power generated by plants on transmission networks, from which the balance (export–import) of exchanges on interconnections between neighboring bidding zones and the power absorbed by energy storage resources is deduced. The total load represents the power demand on the transmission and distribution networks, while any power demand served by distributed networks is not included in the statistics. This aspect influences our measure of the total load, reducing it at times of high generation of renewables in distributed networks. Despite such difference, throughout the paper we refer to load and electricity demand interchangeably.

We estimate future CO2 emissions from electricity generation using technology-specific power generation data recorded on a daily time-step for the 13 European countries included in this study [3] and on a monthly time-step for India's five electricity dispatch regions [10], over the period 2017-2019. The average share of each technology in the regional annual generation mix during 2017-2019 is shown in Table 2. We couple power generation statistics with carbon intensity associated

with the operation of power plants available at the country level for Europe [18] and at the national level for India [14].

Table 2: Share of different technologies in the electricity generation mix (2017-2019 average)

	Gas	Coal	Nuclear	Wind	Solar	Hydro	Other
Europe	34%	27%	20%	8%	2%	6%	3%
India	5%	74%	3%	4%	2%	10%	2%

2 Supplementary Results

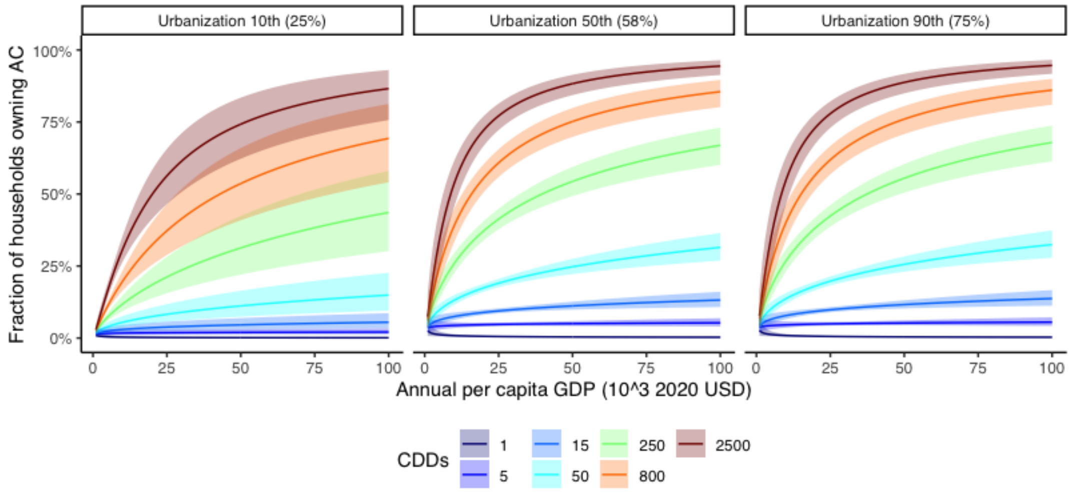

Figure 1: **AC ownership adoption function by urbanization level.** Coloured lines represent the income-AC curves at different levels of exposure to CDDs under the 10th, median and 90th quantile of urbanization level. Coloured shades present the 5th-95th confidence interval of the estimated adoption function.

In order to generate the impacts on electricity demand we rely on 29 GCMs, computing impacts for each model independently and taking the multi-model median of the full sample. We investigate if model selection can influence our results by dividing the full sample of GCMs into two groups, based on the classification proposed by [5]: i) a sub-sample of 13 GCMs providing reasonable projections of warming consistent with the IPCC AR6; a sub-sample of 16 GCMs characterized

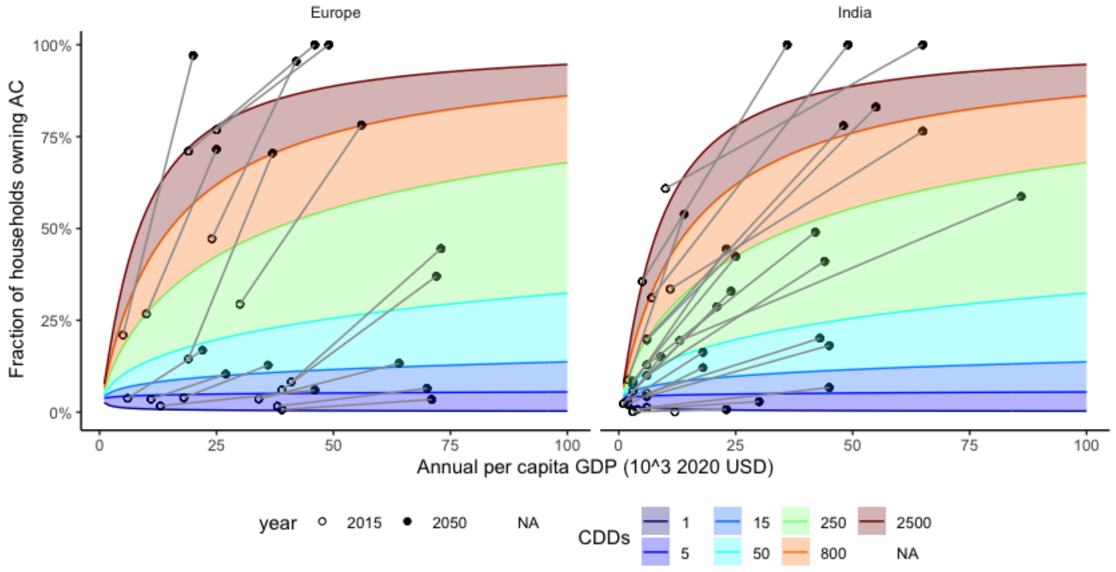

Figure 2: **AC ownership adoption function and projections by state.** Color ranges represent the income-AC curves at different levels of exposure to CDDs under the median urbanization level. Scatters represent the observed state AC ownership rates, as well as the prediction at mid-century under RCP 8.5 and SSP 5. The movements across the coloured areas indicate variations in AC prevalence due to an increase in the exposure to long-run CDDs, while the movements along the coloured areas indicate variations due to higher annual per capita income levels.

by high-sensitivity resulting in "too hot" projections. We find that the ensemble median impact of the "hot" models' group is 18% (10%) higher than the ensemble median impact of the "consistent" models' group in Europe (India), (Table 3). Comparing the projected impacts of each model, we identify only one case in the "hot" group (Tai-ESM1) as a right-end outlier for both India and Europe, and one case in the "AR6 consistent" group (FGOALS-g3) as a left-end outlier for Europe. The distance between the right-end outlier model and the ensemble median is larger for India than for Europe, pointing to regionally heterogeneous differences between GCM projections. The upper (lower) cutoff above (below) which we identify outliers is computed as the sum (difference) between the third (first) quartile and 1.5 times the interquartile range of the full sample (Figure S8, panel b).

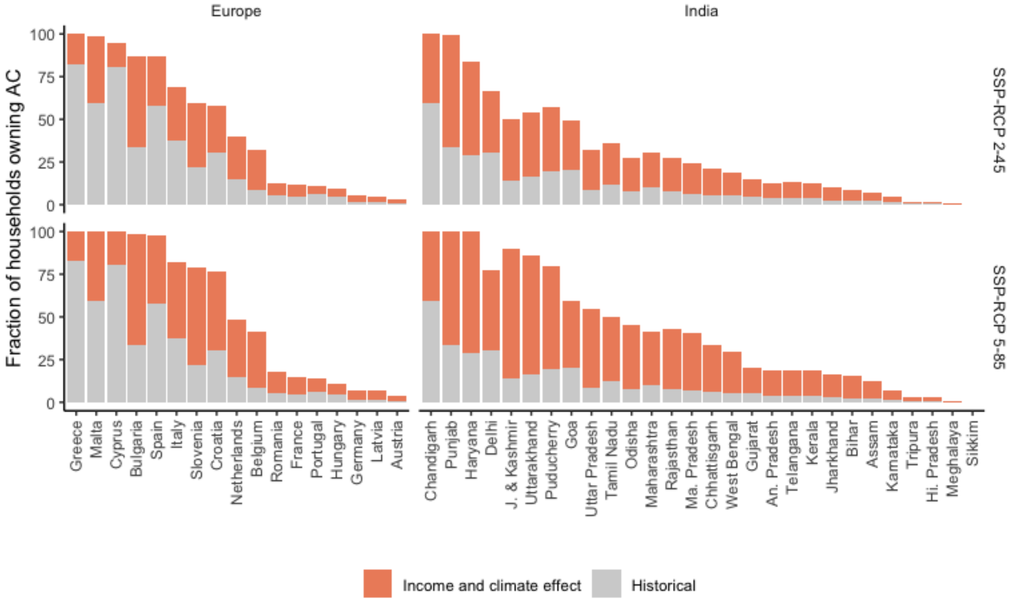

Figure 3: **AC prevalence projection by state and climate scenario** The grey bars present the historical (2015) observed level of AC prevalence, while the coloured bars present the projected increment in AC prevalence due to both income and climate drivers.

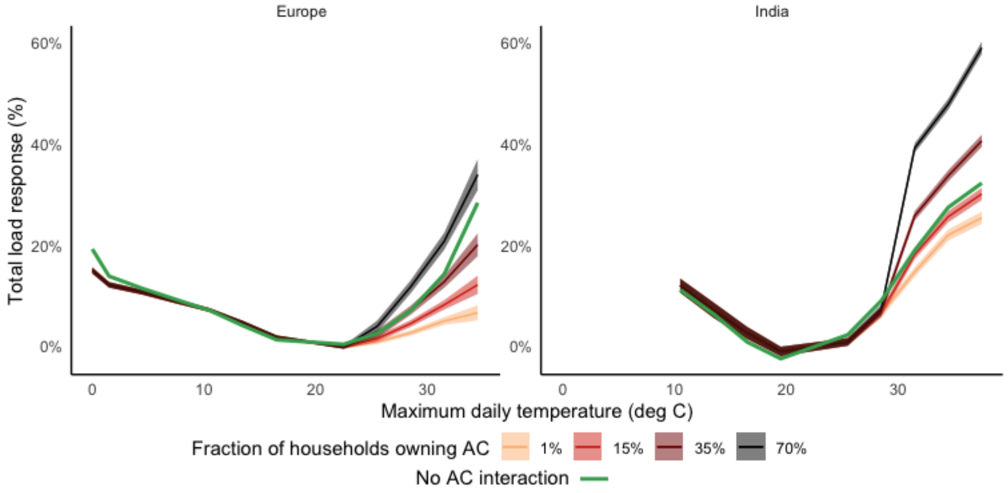

Figure 4: **Macro-regional response of per capita daily total load to maximum daily temperatures.** The coloured shades represent the 95% C.I. of the coefficients estimated based on Equation 4 (see Methods). Vertical bars show the increment in the response under different AC prevalence levels, with respect to the response when AC prevalence equals 1%, in the highest daily temperature interval.

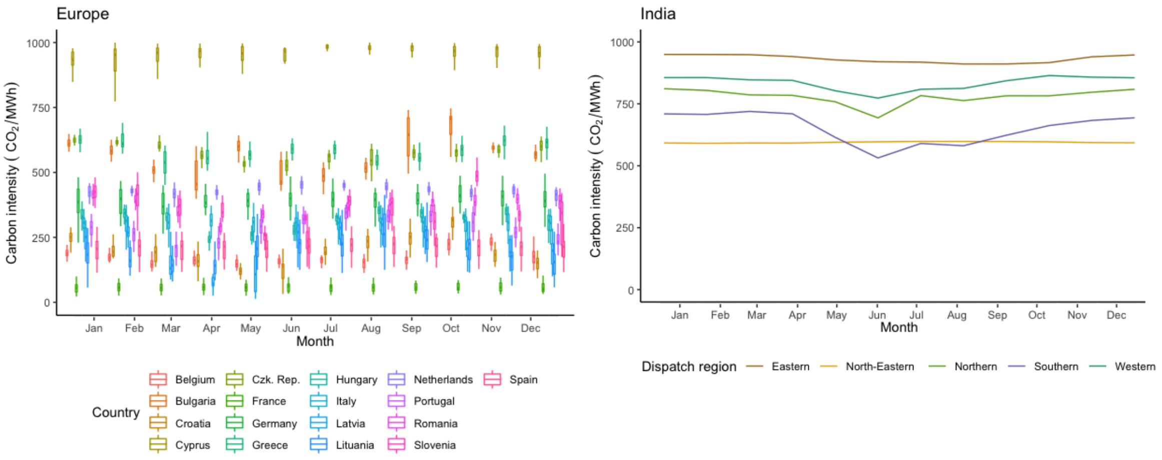

Figure 5: **Seasonal carbon intensity of power generation by state-region.**

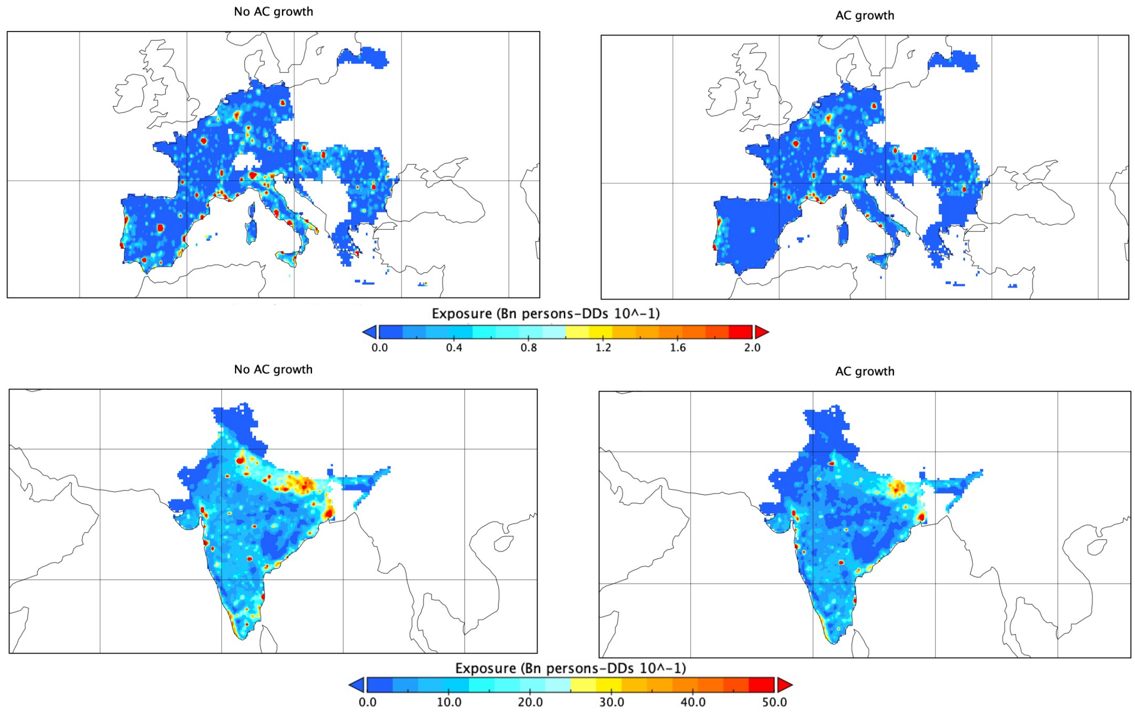

Figure 6: Annual number of people that circa 2050 will be exposed to maximum temperatures above 24°C and have no AC in their homes, measured by the count of person-degree days (DDs), when AC prevalence is fixed to today's level ("no AC growth"), and when extensive margin adjustments allow to increase AC prevalence ("AC growth"). This figure was generated with the open source software Panoply, version 5.2, available at: https://www.giss.nasa.gov/tools/panoply/

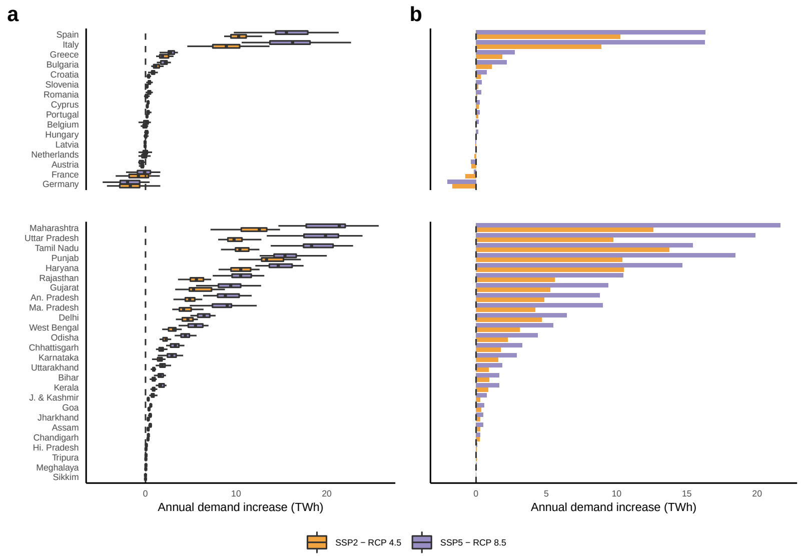

Figure 7: **Electricity demand increase due to climate change circa 2050 from intensive and extensive margins combined by SSP-RCP. a** Relative increase in the annual total load across 29 GCMs. **b** Absolute median increase in the annual total load.

Table 3: Additional annual electricity demand in Europe and India combined, ensemble median impact by group of GCM (TWh)

Europe							
Quantile	min	10%	25%	50%	75%	90%	max
All models	2	24	27	35	40	45	73
AR6 consistent models	2	22	25	28	42	44	50
Hot models	20	26	32	36	39	48	73
India							
Quantile	min	10%	25%	50%	75%	90%	max
All models	135	149	172	184	202	216	368
AR6 consistent models	145	152	172	182	185	200	212
Hot models	135	152	179	199	212	223	368

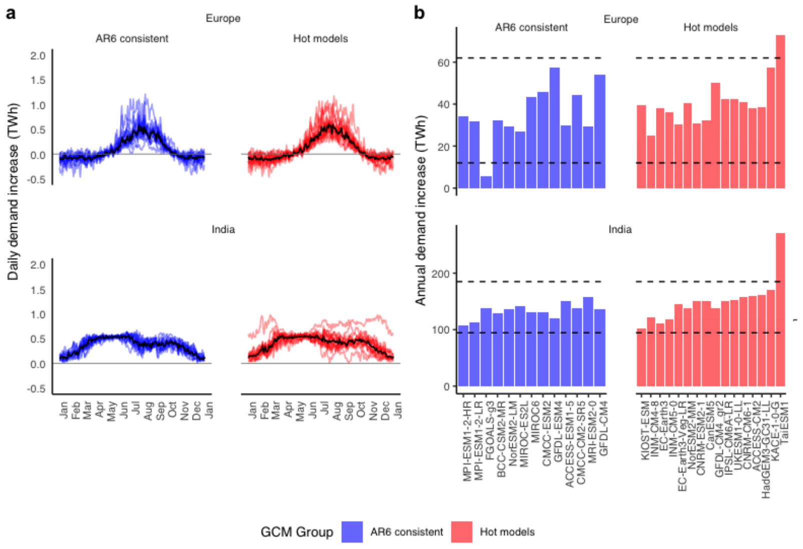

Figure 8: **Impacts on total electricity demand by GCM. a** Daily amplification of electricity demand in each macro-region and GCM, by GCM group. Black lines represent the ensemble-median of each GCM group. **b** Annual amplification of electricity demand in each macro-region and GCM, by GCM group. Black dotted lines represent the upper (lower) cutoff above (below) which we identify outliers, computed as the sum (difference) between the third (first) quartile and 1.5 times the interquartile range of the full sample.

2.1 Energy efficiency and behavioral policies

We compute end-use efficiency improvements based on the regin-specific seasonal energy efficiency ratios (SEERs) available from [1]. We compare the current market average SEER to the best available SEER: the latter is 50% and 40% lower than the former in Europe and India, respectively. We assume that a proportional reduction in the additional electricity demand associated to air-conditioning could be achieved though a shift from current market average to the best available SEER in the two regions.

Table 4: Demand reductions from energy efficiency of appliances

	Average SEER	Best available SEER	Fractional AC demand reduction	Residual demand
Europe	5.5	11	50%	17 TWh
India	3.5	6	41%	109 TWh

We compute the decrease in energy consumption from coupling AC with fans in three steps. First, we identify the potential reduction in air temperature from the operation of fans at different speeds based on the ASHRAE Thermal Environmental Conditions for Human Occupancy [13]. We focus on the case of household occupants undertaking primarily sedentary activity, and select the suggested maximum offset of 3.0°C achieved with air-speed at 0.8 m/s. We compute the new demand amplification circa 2050 (ψ_v^*) by assuming a uniform 3.0°C reduction in air temperatures yielding the exposure to bins ${\mathcal{T}^*}_k^F$, when maximum daily temperatures surpass 24°C.

$$\psi_v^* = \frac{\exp\left[\sum_k \widehat{\beta}_{k,v}^T \widetilde{\mathcal{T}^*}_k^F + \sum_k \widehat{\beta}_{k,v}^{TAC}\left(\widetilde{\mathcal{T}}_k^F \cdot \widetilde{s}^F\right) + \widehat{\beta}_v^Y \widetilde{y}^F\right]}{\exp\left[\sum_k \widehat{\beta}_{k,v}^T \widetilde{\mathcal{T}}_k^C + \sum_k \widehat{\beta}_{k,v}^{TAC}\left(\widetilde{\mathcal{T}}_k^C \cdot \widetilde{s}^C\right) + \widehat{\beta}_v^Y \widetilde{y}^C\right]} \tag{1}$$

Second, we quantify the additional electricity consumption from the use of fans (τ_{i,d^*}) in each state (i) in the days when maximum temperatures surpass 24°C (d^*). Following [8], we assume that each household (h) owning an AC circa 2050 operates a typical 48 inch ceiling fan using 75W of power for a time ranging from 3 to 9 hours/day (r), and that average consumption per hour of operation is 0.075 kWh.

$$\tau_i = \sum_{k,d}(0.075 \cdot r \cdot d_i^* \cdot h_i) \tag{2}$$

Third, we derive the residual demand amplification by taking the sum between ψ_v^* and the additional demand for fans operation (τ_{i,d^*}).

Table 5: Demand reductions from coupling AC and fans

	Fractional AC demand reduction	Additional demand from fans (3-9 hours/day)	Residual demand
Europe	39% - 63%	2 TWh - 7 TWh	16 TWh - 21 TWh
India	50% - 57%	6 TWh - 19 TWh	81 TWh - 94 TWh

References

[1] International Energy Agency. *The Future of Cooling*. 2018.

[2] Central Electricity Authority. *Indian Power Sector Weekly Reports*. 2022.

[3] ENTSO-E. *Transparency Platform*. 2022.

[4] *Eurostat Database*. 2021.

[5] Zeke Hausfather et al. "Climate simulations: recognize the 'hot model'problem". In: *Nature* 605 (2022), pp. 26–29.

[6] Hans Hersbach et al. "The ERA5 global reanalysis". In: *Quarterly Journal of the Royal Meteorological Society* 146.730 (2020), pp. 1999–2049.

[7] Center For Monitoring the Indian Economy. *Consumer Pyramids Database*. 2022.

[8] Arunima Malik et al. "The potential for indoor fans to change air conditioning use while maintaining human thermal comfort during hot weather: an analysis of energy demand and associated greenhouse gas emissions". In: *The Lancet Planetary Health* 6.4 (2022), e301–e309.

[9] Daisuke Murakami, Takahiro Yoshida, and Yoshiki Yamagata. "Gridded GDP Projections Compatible With the Five SSPs (Shared Socioeconomic Pathways)". In: *Frontiers in Built Environment* 7 (2021), p. 760306.

[10] *National Portal of India State Statistics*. 2021.

[11] ODYSSEE-MURE. *Energy Effficiency Database*. 2022.

[12] Niklas Boke Olen and Veiko Lehsten. "High-resolution global population projections dataset developed with CMIP6 RCP and SSP scenarios for year 2010–2100". In: *Data in Brief* 40 (2022), pp. 1–7.

[13] Refrigerating and American National Standards Institute. *Thermal environmental conditions for human occupancy*. Vol. 55. 2004. American Society of Heating, Refrigerating and Air-Conditioning Engineers, 2004.

[14] R.K. Puram Sewa Bhawan. *CO2 Baseline Database for the Indian Power Sector*. 2018.

[15] Bridget Thrasher and Rama Nemani. *NASA Earth Exchange Global Daily Downscaled Projections (NEX-GDDP-CMIP6).* DOI: https://doi.org/10.7917/OFSG3345.

[16] Bridget Thrasher et al. "Bias correcting climate model simulated daily temperature extremes with quantile mapping". In: *Hydrology and Earth System Sciences* 16.9 (2012), pp. 3309–3314.

[17] Bridget Thrasher et al. "NASA Global Daily Downscaled Projections, CMIP6". In: *Nature Scientific Data* ((in review)).

[18] Bo Tranberg et al. "Real-time carbon accounting method for the European electricity markets". In: *Energy Strategy Reviews* 26 (2019), p. 100367.
